# Immobilization of Neutral Protease from *Bacillus subtilis* for Regioselective Hydrolysis of Acetylated Nucleosides: Application to Capecitabine Synthesis

**DOI:** 10.3390/molecules21121621

**Published:** 2016-11-25

**Authors:** Teodora Bavaro, Giulia Cattaneo, Immacolata Serra, Ilaria Benucci, Massimo Pregnolato, Marco Terreni

**Affiliations:** 1 Department of Drug Sciences, University of Pavia, via Taramelli 12, I-27100 Pavia, Italy; teodora.bavaro@unipv.it (T.B.); giulia.cattaneo01@universitadipavia.it (G.C.); massimo.pregnolato@unipv.it (M.P.); 2 Department of Food, Environmental and Nutritional Sciences—DeFENS, University of Milano, via L. Mangiagalli 25, I-20133 Milano, Italy; immacolata.serra@unimi.it; 3 Department for Innovation in Biological, Agro-food and Forest Systems, University of Tuscia, via S. Camillo de Lellis, I-01100 Viterbo, Italy; ilaria.be@unitus.it

**Keywords:** protease N, immobilization, nucleosides, regioselective hydrolysis, anticancer products, capecitabine

## Abstract

This paper describes the immobilization of the neutral protease from *Bacillus subtilis* and its application in the regioselective hydrolysis of acetylated nucleosides, including building blocks useful for the preparation of anticancer products. Regarding the immobilization study, different results have been obtained depending on the immobilization procedure. Epoxy hydrophobic carriers gave a poorly stable derivative that released almost 50% of the immobilized protein under the required reaction conditions. On the contrary, covalent immobilization on a differently activated hydrophilic carrier (agarose) resulted in very stable enzyme derivatives. In an attempt to explain the obtained enzyme immobilization results, the hypothetical localization of lysines on the enzyme surface was predicted in a 3D structure model of *B. subtilis* protease N built in silico by using the structure of *Staphylococcus aureus* metalloproteinase as the template. The immobilized enzyme shown a high regioselectivity in the hydrolysis of different peracetylated nucleosides. A stable enzyme derivative was obtained and successfully used in the development of efficient preparative bioprocesses for the hydrolysis of acetylated nucleosides, giving new intermediates for the synthesis of capecitabine in high yield.

## 1. Introduction

Proteases are hydrolytic enzymes that catalyze the cleavage of proteins. Proteolytic enzymes of bacteria, fungi and viruses are largely studied due to their relevance in biotechnology for industrial applications. Examples of important applications of neutral and alkaline proteases from *Bacillus* sp. have been reported in fermentation and detergent industry [1]; and acid proteases of *Aspergillus* sp. are used in the food industry for the production of cheese [2].

Most of the biotransformations used in food chemistry conveniently employ neutral proteases in soluble form. The use of crude enzyme preparations as catalysts in fine chemical and pharmaceutical processes is however affected by the stability limitations that characterize native enzymes, which could cause product contamination, raising problems of product quality and safety [3], especially in case of drugs for parenteral use, or in the case of several anticancer and antiviral nucleosides.

These drawbacks may be overcome by immobilization of the biocatalyst on a solid carrier; in fact, the binding with a matrix may increase the stability and the catalytic efficiency of the enzyme [4]. Moreover, the solid biocatalysts can be better manipulated than free enzymes. Immobilized enzymes can be recovered and re-used at the end of the reaction, avoiding contamination of the final product with residual proteins. However, immobilization could provide different outcomes depending on the strategy used [5]. For example, immobilization of enzymes normally provides derivatives with better stability, but in many cases a reduction of the enzyme activity is observed (caused by the distortion of the enzyme due to interaction with the support and by diffusional problems). In some instances, the activity may also be enhanced upon immobilization, such as in the case of lipases adsorbed on hydrophobic supports via interfacial activation. However, enzyme adsorption (by ionic or hydrophobic interaction) on solid carriers can be poorly stable and the protein can be released under the non-physiological conditions (pH, temperature or presence of co-solvents) required in preparative processes. On the contrary, covalent immobilization could ensure a suitable stability of the enzyme derivative completely avoiding protein release, with the exception of multimeric enzymes that require an additional post immobilization stabilization for preventing subunit dissociation [6]. In fact, in the case of multimeric enzymes, covalent immobilization would not be suitable to avoid the enzyme inactivation and product contamination. In this case, only one or two subunits can be directly immobilized by covalent attachment on the solid carrier while the others remain free to dissociate. In this case a post-immobilization cross-linking is required for the stabilization of the active multimeric structure of the enzyme, and to avoid the release of the protein from the solid biocatalyst.

Finally, it should be also considered that the catalytic performance of the biocatalyst can be strongly affected by immobilization, depending on the carrier used (nature of the solid matrix and binding chemistry) and the nature of the enzyme [6,7]. Consequently, the selection of the carrier and immobilization condition should be carefully addressed considering physical-chemical phenomena, such as substrate partition and diffusion, that could greatly affect the performance of the immobilized enzyme [8]. In addition, different strategies, including the chemical modification of soluble enzymes or chemical modification of previously immobilized enzymes can be developed for improving the properties of immobilized enzymes [9]. In particular, the micro-environment around the immobilized enzyme as well as any changes induced in its 3D structure by the covalent immobilization process could be the main factors influencing the catalytic properties of immobilized enzymes [10].

Our research group has long studied the enzymatic synthesis of antitumoral and antiviral modified nucleosides. Immobilized lipases have been successfully employed in the regioselective hydrolysis of a large number of peracetylated building blocks [11,12]; however, to obtain an efficient biocatalyst in terms of activity and selectivity, the immobilization of lipases needs to be performed by hydrophobic adsorption [13]. This immobilization presents problems concerning the stability of the enzyme derivative, including the desorption of the enzyme from the carrier in the reactions performed in the presence of co-solvents (as required for the solubilisation of high amounts of substrate).

Enzymes immobilized by covalent binding demonstrated to be viable catalysts in a wide variety of biotransformations, in particular for food and pharmaceutical applications [14,15]. Covalent immobilization can be particularly attractive to improve enzyme stability and to avoid product contamination with residual protein, however, covalent immobilization of lipases provides biocatalysts with very poor activity as a consequence of the reduced flexibility of the 3D structure that prevents the lid to be open for the lipase activation.

Lipases, proteases and esterases have been used as catalysts for the regioselective deprotection of nucleosides, for example, the neutral protease from *B. subtilis* (protease N) was reported to catalyse, in aqueous medium, the selective hydrolysis of the primary position of peracetylated pyrimidine 5-substituted deoxyribonucleosides to give 3′-*O*-acyl counterparts [16]. Furthermore, tri-*O*-acetylated ester of purine and pyrimidine ribonucleosides were selectively converted by using soluble proteases into corresponding nucleosides bearing only a free hydroxyl group in C-5′ position; in all cases, a very high regioselectivity was observed [17,18].

*B. subtilis* secretes at least six extracellular proteases: the neutral proteases A and B, the alkaline protease (subtilisin), extracellular protease, metalloprotease and bacillopeptidase F. Among them, subtilisin and neutral protease A are considered the major proteases since their activity accounts for 20% and 70%, respectively, of total proteolytic activity in the culture medium [19]. Since the composition of the crude preparation named protease N provided by Amano Enzyme and used in the present work, is not clearly defined, we assumed that it mainly consists of neutral protease A (or protease N). This enzyme is one of the most active casein-hydrolysing enzymes reported to date, and it belongs to metalloproteinase class [20,21]. Protease N is the product of *nrpE* gene and, in the mature form, it consists of a polypeptide chain with a molecular weight of approximately 33 kDa [22]. Amino acid sequence is known [19] while 3D structure of this enzyme has not been resolved yet, since the structure determination of *Bacillus* neutral proteases has been hampered by their tendency to autolysis. A large number of papers describe covalent immobilization of hydrolases such as proteases and esterases, for example, trypsin and α-chymotrypsin were recently immobilized on aldehyde- activated carriers by covalent attachment with very good results [23]; on the contrary, only few reports describe the use of immobilized protease N; among them the use of protease N immobilized by adsorption on Celite, as catalyst for peptide bond formation [24].

In the present work, we describe the enzymatic deacetylation of a set of fully acetylated nucleosides **1**–**11**, including two fluorinated compounds **7** and **8**, precursors of capecitabine [25], an important chemotherapeutic agent largely used for the treatment of several cancer forms, including the advanced stage of colon cancer, as well as breast and ovary cancers [26].

All the reactions have been studied by using different hydrolases (esterases, lipases and proteases). The results of this screening show that the highest activity and regioselectivity toward almost all of the substrates considered was obtained with the commercial neutral protease from *B. subtilis*. Therefore, a study for the covalent immobilization of this enzyme by using different carriers and immobilization protocols was performed in order to obtain a derivative with suitable activity and stability useful for developing preparative processes. In particular, the covalent immobilization of this enzyme has been investigated using two commercial carriers characterised by hydrophobic (Eupergit^®^ C and Sepabeads EC-EP) and hydrophilic (agarose) surfaces, pre-activated with epoxy groups and cyanogen bromide (CNBr) respectively. Immobilization can be easily performed directly by reacting these supports with the enzyme at neutral pH, but in this case, a limited number of bonds can be obtained between the carrier and the reactive groups of the protein [27].

Agarose activated with aldehyde groups (glyoxyl) has been also investigated. In this case, the carrier should be activated before the use, but immobilization is performed at high pH to get the immobilization of the enzyme that must be evenly distributed in the first place via several points [28]. Accordingly, this approach allows a multipoint interaction of the enzyme with the carrier that ensure a greater stabilization of the enzyme [15]. Finally, immobilization of Protease N by cross-linking (mediated by glutaraldehyde) has been investigated in order to obtain the immobilization of the protein far from the carrier surface (avoiding protein distortion and the influence of the nature of the carrier). Glutaraldehyde is a reagent frequently used with this goal in order to design efficient biocatalysts [29].

**Scheme 1:**
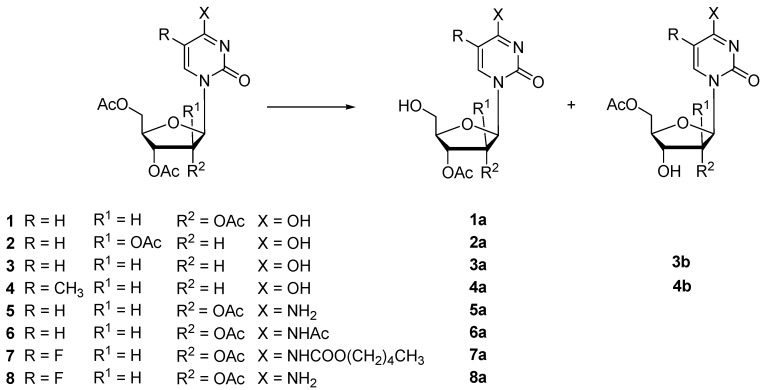
Enzymatic hydrolysis of substrates **1**–**8**.

The effects of the different immobilization procedures on the activity, stability and selectivity of this protease have been evaluated. In particular, the catalytic performance of different biocatalyst preparations was compared with those of the soluble (not immobilized) enzyme in the hydrolysis of peracetylated uridine (**1**) and cytidine (**5**) (Scheme 1) with the aim to prepare 5′-monodeacylated derivatives useful as precursors for different modified nucleosides [11,12]. In order to optimize the experimental conditions, the immobilized enzyme was tested under different temperature and pH conditions, as well as by using organic co-solvents to ensure the complete solubilisation of high concentration of substrates and products.

## 2. Results and Discussion

### 2.1. Screening of Different Hydrolases in the Enzymatic Deacylation of Peracetylated Nucleosides

For a preliminary screening different hydrolyses have been compared in the deacylation of peracetylated nucleosides. The enzymes were used after a simple covalent immobilization on an epoxy carrier (Eupergit^®^ C). The crude extract of *Aspergillus niger* contains several esterases and lipases. In this case, the esterase fraction was immobilized on Eupergit^®^ C after a preliminary removal of the lipases by adsorption on hydrophobic carriers, according to the previously reported procedure [30]. Other biocatalysts were obtained by direct immobilization of the commercial enzyme preparation (see Materials and Methods section).

**Scheme 2:**
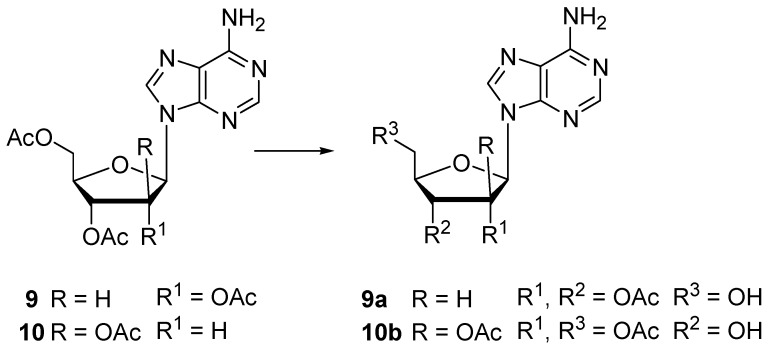
Enzymatic hydrolysis of substrates **9** and **10**.

**Scheme 3:**
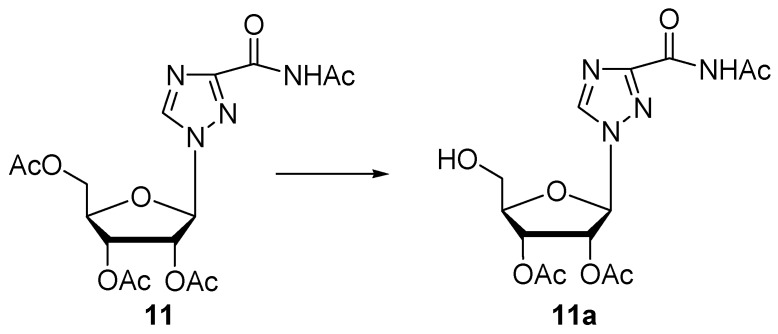
Enzymatic hydrolysis of substrate **11**.

In all cases the immobilization yield (% of activity recovered after complete immobilization of the enzyme) was higher than 20% (results not shown), with the exception of the protease N from *Bacillus subtilis* that provided lower yields (the residual activity was about 20% of the total activity immobilized). The screening was performed with different peracetylated substrates including pyrimidine derivatives (Scheme 1) such as uridine (**1**), arabinosyluracil (**2**), 2′-deoxyuridine (**3**), thymidine (**4**), cytidine (**5**), *N*-acetyl-cytidine (**6**), *N*^4^-pentyloxycarbonyl-5-fluorocytidine (**7**), and 5-fluorocytidine (**8**), as well as some adenosine derivatives including adenosine (**9**), arabinosyladenine (**10**) (Scheme 2) and ribavirin (**11**) (Scheme 3).

In the enzymatic hydrolysis of peracetylated pyrimidine ribonucleosides **1**–**9**, protease N exhibited a marked preference in terms of activity and regioselectivity toward the 5′-position affording the monodeprotected compounds in very good yield (64%–93%), and with a comparable biotransformation rate (Table 1).

The only exception was 2′,3′-*O*-acetylarabinosyluracil (**2a**) synthesized in only 34% yield after 48 h. The yields were much lower compared with those previously reported for *Candida rugose* lipase (CRL), the highest performing enzyme in terms of activity and selectivity toward the 5′-position of pyrimidine nucleosides reported to date [11].

Interestingly, the selectivity of protease N in the hydrolysis of pyrimidine deoxyribonucleosides **3** and **4** was different from those previously reported for CRL [11]; in fact, it was reported that CRL gave a preferential hydrolysis in 3′-position while protease N gives products bearing a free hydroxyl group in position 5′ (**3a** and **4a**) with 74% and 64% yields, respectively [11]. For substrates **5** and **6**, protease N shown similar regioselectivity and afforded similar yields of the products **5a** and **6a** compared with CRL.

**Scheme 4:**
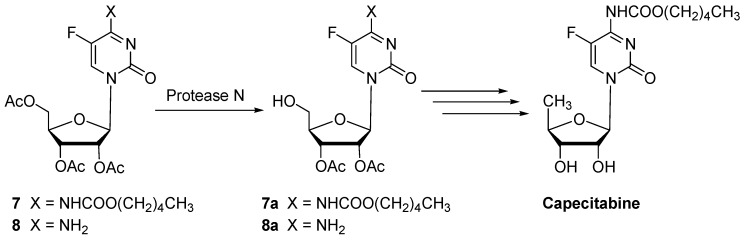
General scheme of capecitabine synthesis.

Protease N was also successfully tested in the hydrolysis of substrates **7** and **8** to give products **7a** and **8a** (in about 90% yield), two new intermediates for the preparation of capecitabine (Scheme 4) [25].

The hydrolysis of the substrates **7** and **8** was also tested with previously reported immobilized CRL [11]. In the case of substrate **8** the results were comparable with those obtained by protease N, while with the substrate **7** the CRL gave a low regioselectivity. In the hydrolysis of substrate **9**, the yield (80%) of the 5′-deprotected product **9a** upon treatment with protease N resulted much higher if compared with those reported for CRL (about 60% of yield) [11]. In the hydrolysis of substrate **10**, the protease from *B. subtilis* shown the same regioselectivity compared with CRL, but in this case, the yield of product **10b** resulted lower than the yield previously reported with the lipase. Generally, a lower regioselectivity than with protease N was observed for esterase from *A. niger* and acylase from *A. melleus* under the same experimental conditions. These enzymes, in fact, gave nearly complete hydrolysis in a shorter reaction time for almost all the substrates tested, if compared with protease N, but the yields of monohydrolysed products were much lower compared with protease N. The esterase from PPL extract shown poor activity and regioselectivity toward the 5′-position of the peracetylated pyrimidine nucleosides.

Finally, the protease N was also tested on peracetylated ribavirin **11**, an example of a base-modified ribonucleoside used for the treatment of hepatitis C infection [31]. Also in this case, the hydrolysis only occurs in position C-5′ in the sugar moiety (31% of **11a** in 48 h), while the hydrolysis of the amide group the heterocycle was not observed (Scheme 3). All the results are summarized in Table 1. Products **1a**–**9a** as well as **10b** and **11a** obtained in the reaction catalysed by protease N, were identified by comparison with analytical standards previously fully characterized by ^1^H and COSY NMR [11,12,25]. Furthermore, product **3b** and **4b**, were not isolated and identified by HPLC analysis of the reaction mixture.

### 2.2. Covalent Immobilization of Protease N

Protease N provided the best results in terms of activity and selectivity in the hydrolysis of the different substrates tested. A better characterization of the derivative obtained by immobilization on epoxy-activated Eupergit C^®^ was performed and this enzyme preparation was compared with other derivatives obtained by immobilization on different carriers. Therefore, the effect induced by the nature of the different carriers (hydrophobic or hydrophilic) and by the use of different immobilization chemistry was evaluated measuring the final activity and stability of the enzyme derivatives prepared.

**Figure 1:**
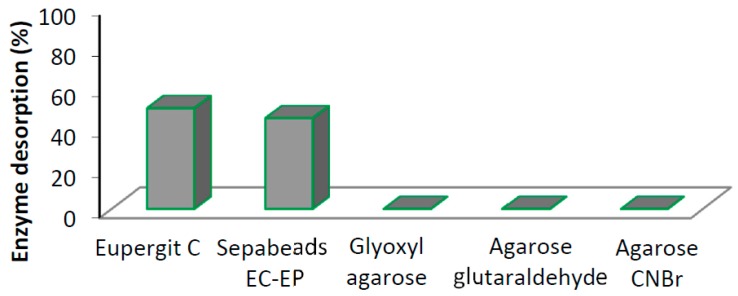
Desorption of protease N in the experimental conditions used for the hydrolysis of different peracetylated nucleosides.

**Figure 2:**
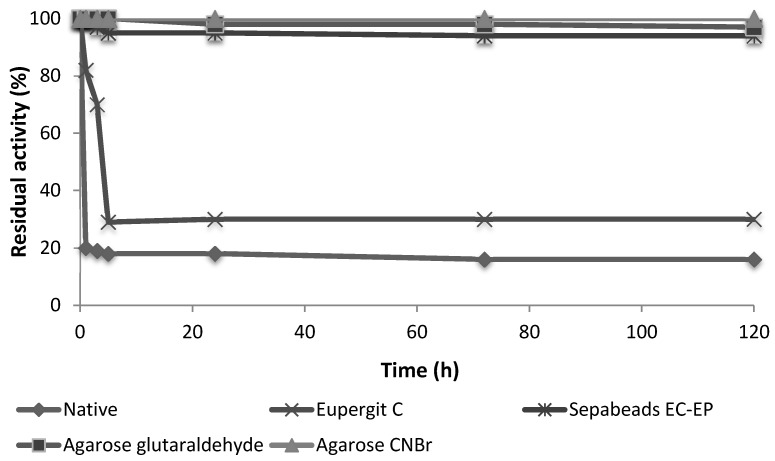
Stability of protease N derivatives in 30% *v*/*v* of acetonitrile. Experimental conditions: 30% CH_3_CN in 25 mM phosphate buffer pH 7, immobilized protease N 250 mg, volume 1 mL, r.t.

First, the stability of native protease N was studied under the experimental conditions required for the different protocols of immobilization considered in this study (Figure S1). In all cases, activity of the soluble enzyme was completely retained after 5 h; after this period, the activity decreased to a different extent depending on the operational conditions. The enzyme is not completely stable under the immobilization conditions used in the case of hydrophobic epoxy carriers such as Eupergit^®^ C and Sepabeads EC-EP. In fact, this immobilization procedure requires 24 h because a first hydrophobic adsorption is induced by using high concentration buffers (at pH 8). Once the enzyme is adsorbed, a covalent interaction occurs between lysine amino groups and the epoxy groups of the carrier [32]. Under these conditions, the enzyme lost more than 50% of its initial activity after 24 h (Figure S1). Accordingly, the final activity obtained after immobilization on epoxy-carriers ranged from 15% to 20% (Table 2). In addition, about 50% of protease N was released from both these catalysts (Figure 1) indicating that not all the loaded protein is covalently linked to the carriers. Therefore, the stability of these enzyme derivatives resulted very low, comparable to the free enzyme (Figure 2). This evidence could explain the low activity and the consequently the long reaction time in the hydrolysis of the different substrates. In order to obtain an active and stable biocatalyst, the covalent attachment of protease N on agarose (a hydrophilic carrier) was investigated using different activations, including activated agarose (glyoxyl-agarose and glutaraldehyde-agarose).

Glyoxyl- and glutaraldehyde agarose possess the same aldehyde functionalization, but in the second case, the reactive groups are placed far from the carrier surface by introduction of a spacer. Glyoxyl activation is directly obtained by oxidation of diols groups on the carrier surface, while the glutaraldehyde carrier is prepared through the reductive amination of the glyoxyl-groups (with ethylenediamine), followed by reaction of the obtained amino groups with glutaraldehyde [33].

In both cases, the immobilization was carried out at high pH values (pH 10) to ensure that the aldehyde groups efficiently react with the amino groups of the enzyme to form the imino double bonds. Imino bonds are reduced with sodium borohydride to obtain irreversible covalent attachment through stable C-N bonds [33].

At a high pH value the amino groups of the enzyme are very reactive and the immobilization is driven by the formation of the first imino double bonds (correlated to the reactivity of the lysines). However, under these conditions, the formation of additional bonds is possible, and a multipoint interaction can be achieved [32]. Immobilization on aldehyde-activated carriers can be also performed at neutral pH but, in this case, a lower interaction degree between the enzyme and the solid matrix would be obtained. Agarose activated with cyanogen bromide (CNBr) groups has been also investigated. This carrier is characterized by the presence of isocyanate groups that directly react with amino groups of the protein. In this case, immobilization efficiently occurs under mild pH conditions [27] forming enzyme derivatives with few interactions between the protein and the carrier (normally the terminal amino group is the only reactive site). The immobilization of protease N on agarose gave similar results regardless of the activation of the carrier (Table 2) and the conditions used, generally, the percentage of expressed activity after immobilization was higher than that achieved on epoxy-hydrophobic carriers. In particular, when glyoxyl agarose was used the immobilization yield (32%) was higher than those obtained by activation with glutaraldehyde (22%). In both cases, the enzyme was completely immobilized and for immobilization at neutral pH on glutaraldehyde-activated carrier the results were similar to those obtained at pH 10 (results not shown). Similarly, immobilization on CNBr agarose was performed at neutral pH, providing a final immobilization yield around 25%–30% (Table 2). No relevant differences in the final activity expressed were observed when the protein loading was increased from 10 to 50 mg/g of carrier.

The immobilization on activated agarose carriers is completed in few hours (see Materials and Methods) and under these conditions, the free enzyme is completely stable (Figure S1). Consequently, the loss of activity observed after immobilization of protease N on agarose, (independently on the experimental conditions and activation of the carrier), seems not correlated to the 3D distortion of the protein structure associated to a high number of covalent bonds with the support. Moreover, the enzyme desorption studies on the differently immobilized protease N shown, in all cases, that enzyme was completely retained by the matrixes (Figure 1). Accordingly, these enzyme derivatives shown a much higher stability than both the free enzyme and the epoxy-carriers derivatives (Figure 2). The stability of the different preparations of protease N was evaluated in presence of buffer/acetonitrile 30% (*v*/*v*) (Figure 2).

As in preparative processes co-solvents could be used to ensure the solubility of substrates, we tested the stability of the derivatives also in the presence of co-solvents [11,12,30]. All the agarose derivatives shown a remarkable stability regardless the activation of the carrier. The retained activity in all cases was complete after 120 h, while the native enzyme and the epoxy-carrier derivatives were rapidly inactivated.

### 2.3. In Silico Evaluation of the 3D Structure of Protease N

In order to explain the results obtained after covalent immobilization of protease N on the different carriers, the hypothetical localization of lysines on the enzyme surface was predicted in a 3D structure model of *B. subtilis* protease N, obtained in silico by using the bioinformatics tools HH-pred [34]; and Modeller [35]. Briefly, HH-pred, starting from a protein query sequence, produces a list of closest homologs. The 3D structural model is then calculated by the MODELLER software from HHpred alignments.

**Figure 3:**
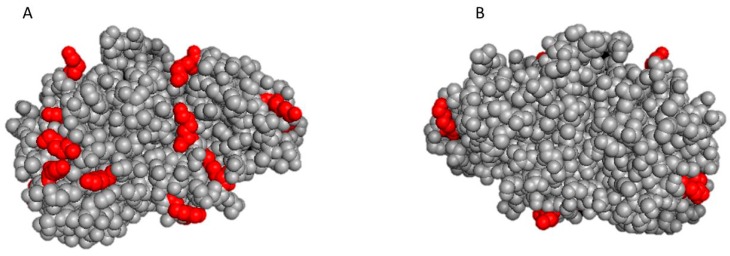
Front (panel **A**) and back side (panel **B**) in the 3D structure of neutral protease A (protease N) from *B. subtilis* built in silico using the structure of *Staphylococcus aureus* metalloproteinase as the template.

In the case of protease N, the amino acid sequence of neutral protease A from *B. subtilis* [21] was used as query, and the closest homolog, with 52% of sequence identity (Figure S2), turned out to be *Staphylococcus aureus* metalloproteinase, which was subsequently used to build the 3D structure model (Figure 3). Since it is not known if protease N exists as a single monomer or as a homodimer, two hypothesis can be considered.

Considering the structure of monomeric protease N, the poor stability of the biocatalyst obtained by immobilization on epoxy carrier of the enzyme can be explained. The simulation of the structure of this enzyme (Figure 3) shown a large side of its surface (around the active site) completely lacking in lysines, differently from the *S. aureus* metalloproteinase used as homolog for the structure prediction and reported in the Supplementary Materials (Figure S3).

During the immobilization on hydrophobic epoxy carrier, the enzyme is very likely adsorbed through this planar area of the enzyme surface. However, after adsorption, the formation of covalent bonds is difficult or almost impossible to occur since the absence of reactive lysines in this side of the protein surface and, consequently, a large surface of the enzyme will be only adsorbed on the carrier, but not covalently linked. On the contrary, the orientation of the enzyme during immobilization on aldehyde or isocyanate activated supports is driven by the formation of the first bonds with the most reactive residues on the protein surface. For this reason, the preliminary adsorption is not necessary and, consequently, the enzyme can be differently oriented with respect to hydrophobic epoxy carriers. Also in the case of a homodimer, immobilization on epoxy hydrophobic carriers will not be suitable to ensure a covalent interaction of the monomers with the support maintaining the intact dimeric active structure of the enzyme. In fact, one protein chain would be linked to the carrier while the other would be free to dissociate thus releasing the 50% of the protein, as observed after immobilization of protease N on epoxy carriers.

Considering what we observed for the monomeric protease, we expected better results for the covalent immobilization of a dimeric enzyme due to the reticular structure of this solid matrix that would ensure the link of both protein chains thus retaining the 3D structure. The possible distortion of the “active” conformation of the dimers could determine a loss of activity after immobilization.

### 2.4. Optimization of Enzymatic Hydrolysis of ***1*** and ***5***

The enzyme preparations obtained with protease N immobilized on agarose activated with glutaraldehyde and agarose CNBr were used as biocatalysts in the hydrolysis of the peracetylated nucleosides **1** and **5** (Scheme 1 and Table 3). The behaviour of these enzyme derivatives was compared with that of the soluble enzyme. The enzymatic hydrolysis was carried out at room temperature in phosphate buffer (pH 7) containing 10% (*v*/*v*) of acetonitrile.

In the hydrolysis of substrate **1** the best results were obtained by using protease N immobilized on agarose CNBr that afforded **1a** in about 94% yield, similarly to what observed with the soluble enzyme. When agarose glutaraldehyde derivative was used as biocatalyst in the hydrolysis of substrate **1**, the yield of **1a** diminished (Table 3).

In the enzymatic hydrolysis of peracetylated cytidine **5**, the higher yield of 5′-monodeprotected compound (**5a**) was obtained with the protease N immobilized on agarose CNBr, compared with the glutaraldehyde derivative. In fact, **5a** was achieved in 66% yield in 48 h using the agarose CNBr derivative, but regioselectivity and yields are lower with respect to the native enzyme (Table 3). The hydrolysis of peracetylated nucleosides **1** and **5** was investigated under different reaction conditions, studying the influence of parameters such as organic co-solvent, temperature or pH. In particular, methanol, acetone and acetonitrile (10% *v*/*v*), were considered as co-solvents.

The temperature has been found to play a pivotal role in controlling the selectivity of the hydrolysis of peracetylated uridine **1**. In fact, as reported in Table 4, when the temperature decreases, the regioselectivity and the yield markedly increased, although a reduction of activity and a consequent increase of the reaction time required for complete hydrolysis of the substrates was observed. In fact, performing the hydrolysis at 4 °C and pH 7, the mono-hydrolysed product **1a** was obtained with 90% yield in 144 h. When peracetylated cytidine **5** was used as substrate, a decrease in the yield of **5a** was observed increasing the pH (Table 4). The yield of **5a** at pH 8.5 was lower with respect to pH 7 (50% instead of 66% yield). The catalytic performance of protease N versus compound **5a** was not affected by temperature (4 °C or at 25 °C) and no difference was observed in the yields obtained. Finally, the best co-solvent resulted acetonitrile, whereas methanol or acetone decreased the yields of all products.

The development of a preparative process revealed a poor productivity by increasing the concentration of peracetylated uridine **1** (Table 5). The yield of **1a** progressively diminished from 94% to 79% when substrate concentration was increased up to 40 mM. On the contrary, the yield of **5a** increased from 66% to 85% when concentration was increased from 10 to 40 mM (Table 5).

## 3. Materials and Methods

### 3.1. General Procedures

The crude extracts of lipases from *Porcine pancreas* (PPL) and *Aspergillus niger* (ANL) were purchased from Sigma-Aldrich (Milano, Italy). The crude extracts of protease N from *Bacillus subtilis* and acylase *Aspergillus melleus* were kindly donated by Amano Enzyme Europe (Chipping Norton, UK). Uridine was kindly donated by Adorkem Technology s.p.a. (Costa Volpino, Bergamo, Italy); arabinosyluracil, 2′-deoxyuridine, adenosine, arabinosyl adenine and peracetylated ribavirin were kindly donated by Pro.bio.sint s.r.l. (Varese, Italy). Protease N PRD0950504N was a gift from Amano Enzyme (Tokyo, Japan). Eupergit^®^ C was kindly donated from Rohmpharma Rohm GmbH (Darmstadt, Germany). Sepabeads EC-EP and EC-HG was a gift from Resindion (Binasco, Milano, Italy). Bradford reagent, cyanogen bromide activated-Sepharose^®^ 4B, *N*_α_-Benzoyl-l-arginine ethyl ester hydrochloride (BAEE), Bovine Serum Albumin (BSA), agarose, cytidine and thymidine were purchased from Sigma-Aldrich.

Chromatographic purifications were performed on silica gel (Merck 60, Munich, Germany, 40–63 µm) with the solvent system indicated; TLC analyses were run on silica plates (Merck 60 F_254_) and visualized by UV light (254 nm). HPLC analyses were run with a L-7100 HPLC (Merck Hitachi, Tokyo, Japan) equipped with Merck Hitachi D-7000 HPLC Multi HSM Manager, a L-7400 detector, an L-7300 oven and a C18 column (Kromasil, 250 mm × 4.6 mm, 5 µm particles) The analysis were conducted at 1 mL·min^−1^. UV wavelength and the percentage of organic modifier are selected according to the maximum of absorption of the desired compound. The pH during the enzymatic hydrolyses was kept constant by automatic titration and the enzymatic activities were measured by using a pH-stat 718 Stat Tritino (Metrohm, Herisau, Switzerland). Peracetylated nucleosides **1**–**6** and **8**,**9** were synthesized using well-known previously reported methods [11]. Compound **7** was prepared in according to the literature [25]. Products **1a**–**9a** as well as **10b** and **11a** obtained in the reaction catalysed by protease N, were identified by comparison with analytical standards previously fully characterized by ^1^H and COSY NMR [11,12,25]. Furthermore, product **3b** and **4b**, were not isolated and identified by HPLC analysis of the reaction mixture.

### 3.2. Enzyme Immobilization

Enzyme immobilization on different supports was realized applying the respective immobilization procedure (see below), the enzymatic activity was monitored during the immobilization by enzymatic standard assay and the amount of immobilized enzyme was assessed by measuring the enzyme concentration before and after immobilization (Bradford method). The enzymatic preparation was washed with deionized water and kept at 4 °C whenever not in use.

#### 3.2.1. Immobilization of Esterase Fraction Contained in the Crude Extract of *Aspergillus niger* Lipase

The crude extract of ANL (1.6 g), containing three lipase fractions (31, 43 and 65 kDa), was dissolved in 22 mL of 10 mm phosphate buffer pH 7. The solution was stirred at room temperature for 1 h. Octyl agarose (4 g), previously washed with the same buffer, was added to the enzyme solution and the suspension was kept under stirring for 1.5 h at 4 °C. The octyl agarose derivative was then filtered and washed with distilled water. To the solution was then added octadecyl Sepabeads (2 g), previously washed with the same buffer, and the suspension was kept under stirring for 1.5 h at 4 °C. The enzyme derivative, containing a lipase fraction (31 kDa), was then filtered and washed with distilled water. The supernatant, showing esterase activity, was diluted 1:1 with a solution of 2 M phosphate buffer at pH 7.5. Sepabeads EC-EP (4 g), previously washed with the same buffer, was added and the suspension was stirred at room temperature for 24 h. The enzyme derivative was then filtered and washed with distilled water.

#### 3.2.2. Immobilization of Esterase PPL and Acylase on Eupergit^®^ C

The crude extract (0.5 g) were dissolved in potassium phosphate buffer 1 M pH 8.0 (12.6 mL). After, 1 g of support, previously washed with water and potassium phosphate buffer, was added thereto. The suspension was kept under stirring for 24 h at room temperature and then filtered. The enzymatic preparation was washed with deionized water and kept at 4 °C whenever not in use.

#### 3.2.3. Immobilization of Protease N on Eupergit^®^ C and Sepabeads EC-EP

25 IU of protease N crude extract (0.5 g) were dissolved in potassium phosphate buffer 1 M pH 8.0 (12.6 mL). After, 1 g of support, previously washed with water and potassium phosphate buffer, was added thereto. The suspension was kept under stirring for 24 h at room temperature and then filtered. The enzymatic preparation was washed with deionized water and kept at 4 °C whenever not in use.

#### 3.2.4. Immobilization of Protease N on Glyoxyl Agarose and Agarose Glutaraldehyde

The supports were previously activated with the respective procedure and then submitted to enzyme immobilization. The glyoxyl-agarose is obtained by reaction of agarose gel in reductive-basic environment (NaOH 1.7 M, with NaBH_4_ 28.4 mg/mL, 4 °C) with glycidol overnight, then oxidized with NaIO_4_ for 2 h and washed.

The agarose glutaraldehyde support was activated as previously reported [34], briefly the aldehyde-agarose (17.5 g) is aminated with ethylenediamine (EDA, 2 M pH 10.00) for 2 h and reduced with NaBH_4_ (1 g, 2 h). The EDA activated agarose was then suspended in 0.2 M phosphate buffer pH 7 (3.4 mL) and a solution of 25% (*v*/*v*) glutaraldehyde (5.1 mL) was added. The mixture was kept under stirring for 16 h at room temperature in the darkness. 

Immobilization on aldehyde activated carriers was performed slightly modifying the procedure previously reported [36,37]. The aldehyde-agarose gel (1.4 mL), glyoxyl agarose or agarose gluteraldehyde, were suspended in 50 mM carbonate buffer at pH 10.05. After the addition of 25 IU of protease N enzyme extract (0.5 g), the suspension (14 mL) was kept under mechanical stirring during 2.5 h. The chemical reduction of Schiff bases was carried out by adding to the mixture 14 mg of NaBH_4_ (1 mg/mL) over 30 min. The immobilized enzyme was then filtered and washed with 10 mM potassium phosphate buffer pH 5.0. 

#### 3.2.5. Immobilization of Protease N on Cyanogen Bromide-Activated Sepharose^®^ 4B

25 IU of protease N (0.5 g of crude extract) were dissolved in 12.6 mL of immobilization buffer (0.1 M NaHCO_3_, 0.5 M NaCl pH 8.5). The support (1 g) was previously activated in 200 mL of 1 mM HCl solution for 15 min, after that it was filtered and conditioned with immobilization buffer and added to the enzymatic solution (final volume 14 mL). The suspension was kept at room temperature under stirring for 2.5 h and then filtered. the immobilization requires a quenching passage realized for 2 h at 4 °C under stirring in 40 mL of 0.1 M TRIS-HCl buffer pH 8.0.

### 3.3. Activity Assay

To a solution of *N*_α_-benzoyl-l-arginine ethyl ester hydrochloride (BAEE, 17 mg) in 25 mM phosphate buffer pH 7 (final volume = 5 mL, (BAEE) = 10 mM), the enzyme was added (10 mg of protease N enzymatic extract or 10–50 mg of immobilized enzyme). The reaction was kept under mechanical stirring at room temperature. At fixed times, aliquots of the reaction mixture were withdrawn (20 and 30 min), filtered by centrifugation (10 kDa MWCO centrifugal filter devices, 12,000 rpm, 22 °C, 10 min) to remove the protein and analyzed by HPLC using the method below reported for the separation of the product, *N*_α_-benzoyl-l-arginine acid (BAA), from the unreacted substrate Scheme 1). Analytical method: A: 10 mM potassium phosphate buffer pH 4.2 (75%, *v*/*v*), B: 9:1 CH_3_CN/H_2_O (25%, *v*/*v*) isocratic elution, λ 223 nm, R_t__BAA_ = 3.1 min, R_t__BAEE_ = 7.0 min. The enzyme activity was calculated as IU, that corresponds to the amount of enzyme that converts 1 µmoL of BAEE into BAA per minute.

### 3.4. Enzyme Stability

The enzyme stability was evaluated in phosphate buffer (25 mM, pH 7.0) in presence of 30% (*v*/*v*) of CH_3_CN during 5 days period at 25 °C. The samples were periodically withdrawn and their activities were measured with the activity assay described elsewhere.

### 3.5. Evaluation of the Attachment between the Enzyme and the Support

The different enzyme derivatives were incubated with buffered solutions containing acetonitrile 10% (*v*/*v*) at pH 7 for 24 h. After that, the release of the protein in the supernatant was determined according to Bradford assay [38] using bovine serum albumin as standard. 

### 3.6. Enzymatic Hydrolysis of Peracetylated Nucleosides ***1**–**10***

A solution of a peracetylated nucleoside (10–40 mM) in acetonitrile 10% (*v*/*v*) was added to a solution of potassium phosphate buffer (pH 7, 25 mM). The pH was adjusted to 7.0 and the appropriate amount of immobilized protease N was added. The suspension was maintained under mechanical stirring at room temperature until the maximum hydrolysis of the substrate. During the reaction the pH was kept constant by automatic titration (Metrohm 718 STAT Tritino). Samples of the reaction mixture were analyzed at different times by TLC and HPLC. Finally, the enzyme was filtered off and washed with deionized water and a solution of acetonitrile (10%), and the filtrate was concentrated under reduced pressure and extracted with ethyl acetate (3 × 20 mL). The collected organic phases were dried with anhydrous Na_2_SO_4_, filtered, and dried under vacuum. The residue, when necessary, was further purified by silica gel column chromatography (CH_2_Cl_2_ 100% to CH_2_Cl_2_/MeOH, 97:3) to afford the different deprotected nucleosides **1a**–**9a**, **3b**, **4b** and **10b**.

### 3.7. Semi-preparative Method

Hydrolysis reaction was purified by using an HP-1100 pump (Agilent, Palo Alto, CA, USA), equipped with a manual Rheodyne sample valve (1.4 mL loop) and connected to octadecyl silica columns (LichroCart C18, 250 mm × 10 mm, 10 µm) and a single wavelength UV-detector (λ 260 nm). The system was connected to an HPLC ChemStation (Revision A.04.01). The analytical method was adapted to semi-preparative purpose: the flow rate was enhanced to 4 mL·min^−1^ and the proper volatile buffer have been chosen. Mobile phases were A: 100% 10 mM ammonium acetate buffer where pH was adjusted to 4.2 adding acetic acid, B: 9:1 CH_3_CN/H_2_O and the purification was realized with 30% of B in isocratic mode and peaks were collected separately. Identity and purity of each fraction was identified by injection on the analytical system. After the identification, the solution corresponding to the desired product was lyophilised.

## 4. Conclusions

In this work, the protease N from *B. subtilis* has been conveniently immobilized on agarose (hydrophilic carrier) by covalent attachment independently from the activation of the carrier, maintaining about the 30% of initial activity and producing solid biocatalysts completely stable in presence of co-solvent for the regioselective hydrolysis of acetylated nucleosides. Differently, the immobilization on an hydrophobic acrylic carrier activated with epoxy groups leads to instable biocatalysts because part of the enzyme is released from the support under the reaction conditions. The enzyme leakage seems related to the fact that part of the protein is not covalently linked to the carrier, but it is only adsorbed in its monomeric or dimeric form. This adsorption seems mediated by a large area of the protein surface lacking in lysines (the amino acid reactive in the covalent interaction).

Protease N gave similar or better results compared with immobilized CRL, the best catalyst reported to be a useful catalyst for the hydrolysis of peracetylated nucleosides (substrates **1**, **5**, **6** and **9**), to obtain products with a free hydroxyl group in position C-5′. For this reason, the biocatalysts have been tested also in the hydrolysis of two derivatives of the fluorinated cytosine in order to obtain products **7a** and **8a**, useful as building blocks for the synthesis of Capecitabine and, in both cases, immobilized protease N provided better results compared with CRL. Interestingly, the selectivity of protease N in the hydrolysis of deoxyribonucleosides **3** and **4** was opposite compared with the results previously reported for CRL allowing the hydrolysis in position C-5′ instead of C-3′.

Finally, in the hydrolysis of arabinosylnucleosides (substrates **2** and **10**), the protease N maintain the same regioselectivity previously observed with CRL, but afforded much lower yields of products **2a** and **10b** compared with this lipase.

In conclusion, the protease N covalently immobilized on agarose can be preferred to the immobilized CRL for the preparation of acetylated ribonucleosides with only one free hydroxyl group in position C-5′, as useful building blocks for the synthesis of monophosphate nucleotides or 5′-deoxyribonucleosides such as Capecitabine. The use of agarose-CNBr can be conveniently used for the preparation of a solid biocatalyst suitable to be used in preparative reactions. Accordingly, with the protease N derivative acetylated nucleosides bearing only one free hydroxyl group have been prepared in 80%–85% yield and up to 14 g/L of concentration.

## Figures and Tables

**Table 1:** Enzymatic hydrolysis of peracetylated pyrimidine nucleosides **1**–**11**.

Substrate	Enzyme	t (h)	Vh ^1^	Conversion (%)	Products (Yield %)		
					5′-OH	3′-OH
**1**	Protease N	22	0.52	100	**1a** (93)	n.i.
	Esterase PPL	96	0.02	79	**1a** (52)	n.i.
	Esterase ANL	5	3.13	98	**1a** (20)	n.i.
	Acylase	4	0.66	97	**1a** (56)	n.i.
**2**	Protease N	48	0.04	48	**2a** (34)	n.i.
	Esterase PPL	120	0.02	47	**2a** (28)	n.i.
	Esterase ANL	5	5.66	99	**2a** (35)	n.i.
	Acylase	24	0.10	97	**2a** (14)	n.i.
**3**	Protease N	48	0.10	93	**3a** (64)	**3b** (13)
	Esterase PPL	48	0.01	28	**3a** (23)	**3b** (5)
	Esterase ANL	5	4.46	99	**3a** (44)	**3b** (2)
	Acylase	5	0.68	98	**3a** (49)	**3b** (5)
**4**	Protease N	24	0.71	98	**4a** (74)	**4b** (6)
	Esterase PPL	48	0.36	91	**4a** (41)	**4b** (8)
	Esterase ANL	3	2.07	92	**4a** (42)	**4b** (10)
	Acylase	5	0.07	92	**4a** (68)	**4b** (9)
**5**	Protease N	48	0.16	94	**5a** (84)	n.i.
	Esterase PPL	48	0.90	88	**5a** (51)	n.i.
	Esterase ANL	5	5.10	98	**5a** (13)	n.i.
	Acylase	3	0.89	97	**5a** (49)	n.i.
**6**	Protease N	48	0.19	82	**6a** (77)	n.i.
	Esterase PPL	48	0.09	99	**6a** (54)	n.i.
	Esterase ANL	3	6.14	99	**6a** (22)	n.i.
	Acylase	7	0.35	94	**6a** (73)	n.i.
**7**	Protease N	24	0.10	97	**7a** (92)	n.i.
	Esterase PPL	24	0.15	92	**7a** (48)	n.i.
	Esterase ANL	5	4.30	94	**7a** (66)	n.i.
	Acylase	18	0.18	90	**7a** (78)	n.i.
	CRL	3	3.3	98	**7a** (39)	n.i.
**8**	Protease N	30	0.18	99	**8a** (89)	n.i.
	CRL	30	0.12	93	**8a** (80)	n.i.
**9**	Protease N	48	0.12	93	**9a** (80)	n.i.
	Esterase PPL	48	0.03	72	**9a** (72)	n.i.
	Esterase ANL	3	4.42	97	**9a** (21)	n.i.
	Acylase	3	1.11	99	**9a** (42)	n.i.
**10**	Protease N	24	0.09	55	n.i.	**10b** (42)
	Esterase PPL	24	0.04	36	n.i.	**10b** (34)
	Esterase ANL	5	3.61	99	n.i.	**10b** (57)
	Acylase	4	0.77	99	n.i.	**10b** (12)
**11**	Protease N	48	0.05	62	**11a** (31)	n.i.
	Esterase PPL	48	0.10	58	**11a** (31)	n.i.
	Esterase ANL	4	3.16	98	**11a** (26)	n.i.
	Acylase	4	0.76	100	**11a** (3)	n.i.

Experimental conditions: 10% CH_3_CN in 25 mM KH_2_PO_4_ buffer pH 7, immobilized enzyme 50 IU, reaction volume 2.5 mL, (substrate): 10 mM, r.t., n.i.: not isolated, ^1^ μmol∙min/IU.

**Table 2:** Covalent immobilization of protease N.

Support	Activation	Loading ^1^ mg Protein/g Support	Imm. Protein ^2^ (%) (SD)	Activity ^3^ (IU·g^−1^) (SD)	Yield ^4^ (%) (SD)
Eupergit^®^ C	Epoxy	10	62 (8)	1 (0.5)	20 (10)
Sepabeads EC-EP	Epoxy	10	58 (11)	0.8 (0.4)	16 (8)
Glyoxyl agarose	Aldehyde	10	90 (6)	1.6 (0.3)	32 (6)
Agarose glutaraldehyde	Aldehyde	10	98 (4)	1.1 (0.2)	22 (4)
Agarose CNBr	Isocyanate	10	96 (3)	1.2 (0.3)	24 (6)
Agarose CNBr	Isocyanate	50	65 (5)	7.8 (0.4)	31 (2)

^1^ Loaded activity (IU*·*g^−1^): 5 IU for a loading of 10 mg/g, 25 IU for a loading of 50 mg/g (IU = µmol of substrate hydrolyzed per min), ^2^ Measured by Bradford assay, ^3^ The activity of immobilized protease N is expressed as IU·g^−1^ of support, ^4^ (Activity of immobilized protease N/loaded activity) × 100, SD = standard deviation.

**Table 3:** Enzymatic screening of peracetylated nucleosides (**1**, **5**) by immobilized protease N.

Substrate	Support	t (h)	Vh	Conversion (%)	Product (Yield %) 5′-OH
**1**	Native enzyme	22	0.52	100	**1a** (93)
**1**	Agarose CNBr	15	0.14	99	**1a** (94)
**1**	Agarose glutaraldehyde	24	0.14	81	**1a** (69)
**5**	Native enzyme	48	0.16	92	**5a** (84)
**5**	Agarose CNBr	48	0.05	94	**5a** (66)
**5**	Agarose glutaraldehyde	216	0.06	88	**5a** (54)

Experimental conditions: 10% CH_3_CN in 25 mM phosphate buffer pH 7, immobilized protease N 2 IU, reaction volume 10 mL, substrate: 10 mM, r.t.

**Table 4:** Enzymatic hydrolysis of peracetylated nucleosides **1** and **5** catalysed by agarose CNBr-protease N.

Substrate	Solvent	pH Temperature	t (h)	Vh ^1^	Conversion (%)	Product (Yield %), 5′-OH
**1**	MeOH	pH 7, 25 °C	7	0.12	96	**1a** (74)
**1**	Acetone	pH 7, 25 °C	23	0.09	99	**1a** (82)
**1**	CH_3_CN	pH 7, 25 °C	15	0.14	99	**1a** (94)
**1**	CH_3_CN	pH 7, 4 °C	144	0.02	99	**1a** (90)
**1**	CH_3_CN	pH 5.5, 25 °C	216	0.01	95	**1a** (82)
**1**	CH_3_CN	pH 8.5, 25 °C	27	0.08	97	**1a** (72)
**5**	MeOH	pH 7, 25 °C	48	0.07	88	**5a** (42)
**5**	Acetone	pH 7, 25 °C	120	0.02	85	**5a** (58)
**5**	CH_3_CN	pH 7, 25 °C	48	0.09	94	**5a** (66)
**5**	CH_3_CN	pH 7, 4 °C	360	0.01	82	**5a** (63)
**5**	CH_3_CN	pH 5.5, 25 °C	192	0.005	92	**5a** (62)
**5**	CH_3_CN	pH 8.5, 25 °C	120	0.02	93	**5a** (50)

Experimental conditions: 10% of solvent in 25 mM phosphate buffer, immobilized lipase 2 IU, reaction volume 1.25 mL, (substrate): 10 mM, ^1^ μmol∙min/IU.

**Table 5:** Hydrolysis of substrates **1** and **5** at different concentration.

Substrate	Conc. (mM)	t (h)	Vh ^1^	Conversion (%) (SD)	Product (Yield %) 5′-OH (SD)
**1**	10	24	0.48	94 (5)	**1a** (94) (5)
**1**	20	48	0.30	92 (4)	**1a** (86) (6)
**1**	40	72	0.28	90 (8)	**1a** (79) (10)
**5**	10	48	0.09	94 (3)	**5a** (66) (7)
**5**	40	72	-	88 (9)	**5a** (85) (6)

Experimental conditions: 10% CH_3_CN in 25 mM phosphate buffer pH 7, agarose CNBr-protease N 6 IU, reaction volume 5 mL, r.t., SD = standard deviation.

## Supplementary Materials

Supplementary materials can be accessed at: http://www.mdpi.com/1420-3049/21/12/1621/s1.

## References

[1] Bott R. (1997). Development of new proteases for detergents. Surfactant Sci. Ser..

[2] Sumantha A., Larroche C., Pandey A. (2006). Microbiology and industrial biotechnology of food-grade proteases: A perspective. Food Technol. Biotechnol..

[3] Tufvesson P., Lima-Ramos J., Nordblad M., Woodley J.M. (2011). Guidelines and cost analysis for catalyst production in biocatalytic processes. Org. Process Res. Dev..

[4] Cao L. (2005). Immobilised enzymes: Science or art?. Curr. Opin. Chem. Biol..

[5] Rodrigues R.C., Ortiz C., Berenguer-Murcia A., Torres R., Fernández-Lafuente R. (2013). Modifying enzyme activity and selectivity by immobilization. Chem. Soc. Rev..

[6] Fernandez-Lafuente R. (2009). Stabilization of multimeric enzymes: Strategies to prevent sub-unit dissociation. Enzym. Microb. Technol..

[7] Guzik U., Hupert-Kocurek K., Wojcieszynska D. (2014). Immobilization as a strategy for improving enzyme properties-application to oxidoreductases. Molecules.

[8] Cantone S., Ferrario V., Corici L., Ebert C., Fattor D., Spizzo P., Gardossi L. (2013). Efficient immobilisation of industrial biocatalysts: Criteria and constraints for the selection of organic polymeric carriers and immobilisation methods. Chem. Soc. Rev..

[9] Rodrigues R.C., Berenguer-Murcia Á., Fernandez-Lafuente R. (2011). Coupling chemical modification and immobilization to improve the catalytic performance of enzymes. Adv. Synth. Catal..

[10] Bonomi P., Bavaro T., Serra I., Tagliani A., Terreni M., Ubiali D. (2013). Modulation of the microenvironment surrounding the active site of Penicillin G Acylase immobilized on acrylic carriers improves the enzymatic synthesis of cephalosporins. Molecules.

[11] Bavaro T., Rocchietti S., Ubiali D., Filice M., Terreni M., Pregnolato M. (2009). A versatile synthesis of 5’-functionalized nucleosides through regioselective enzymatic hydrolysis of their peracetylated precursors. Eur. J. Org. Chem..

[12] Bavaro T., Ubiali D., Brocca S., Rocchietti S., Nieto I., Pregnolato M., Lotti M., Terreni M. (2010). Recombinant lipase from *Candida rugosa* for regioselective hydrolysis of peracetylated nucleosides. A comparison with commercial non recombinant lipases. Biocatal. Biotransf..

[13] Bastida A., Sabuquillo P., Armisen P., Fernàndez-Lafuente R., Huguet J., Guisàn J.M. (1998). A single step purification, immobilization, and hyperactivation of lipases via interfacial adsorption on strongly hydrophobic supports. Biotechnol. Bioeng..

[14] Guisan J.M., Betancor L., Fernandez-Lorente G. (2009). Encyclopedia of Industrial Biotechnology: Bioprocess, Bioseparation, and Cell Technology.

[15] Mateo C., Palomo J.M., Fuentes M., Betancor L., Grazu V., López-Gallego F., Pessela B.C.C., Hidalgo A., Fernández-Lorente G., Fernández-Lafuente R. (2006). Glyoxyl agarose: A fully inert and hydrophilic support for immobilization and high stabilization of proteins. Enzym. Microb. Technol..

[16] Uemura A., Nozaki K., Yamashita J., Yasumoto M. (1989). Lipase-catalyzed regioselective acylation of sugar moieties of nucleosides. Tetrahedron Lett..

[17] Haribansh K., Singh G., Cote L., Sikors S.R. (1993). Enzymatic regioselective deacylation of 2′,3′,5′-tri-*O*-acylribonucleosides: Enzymatic synthesis of 2′,3′-di-*O*-acylribonucleosides. Tetrahedron Lett..

[18] Panero J., Trelles J., Rodano V., Montserrat J.M., Iglesias L.E., Lewkowicz E.S., Iribarren A.M. (2006). Microbial hydrolysis of acetylated nucleosides. Biotechnol. Lett..

[19] Tran L., Wu X., Wong S. (1991). Cloning and expression of a novel protease gene encoding an extracellular neutral protease from *Bacillus subtilis*. J. Bacteriol..

[20] Mcconn J.D., Tsuru D., Yasunobu K.T. (1964). *Bacillus subtilis* neutral proteinase. I. A zinc enzyme of high specific. J. Biol. Chem..

[21] Tsuru D., Mcconn J.D., Yasunobu K.T. (1965). *Bacillus subtilis* neutral proteinase. II. Some physicochemical. J. Biol. Chem..

[22] Yang M.Y., Ferrari E., Henner D.J. (1984). Cloning of the neutral protease gene of *Bacillus subtilis* and the use of the cloned gene to create an in vitro-derived deletion mutation. J. Bacteriol..

[23] Marques D., Pessela B.C., Betancor L., Monti R., Carrascosa A.V., Rocha-Martin J., Guisan J.M., Fernandez-Lorente G. (2011). Protein hydrolysis by immobilized and stabilized trypsin. Biotechnol. Prog..

[24] Miyazawa T., Masaki S., Tanaka K., Yamada T. (2003). Peptide syntheses mediated by *Bacillus subtilis* protease. Lett. Pept. Sci..

[25] Pregnolato M., Terreni M., Ubiali D., Bavaro T. (2008). 2′,3′-Di-*O*-Acyl-5-fluoronucleosides. Patent.

[26] Di Costanzo F., Sdrobolini A., Gasperoni S. (2000). Capecitabine, a new oral fluoropyrimidine for the treatment of colorectal cancer. Crit. Rev. Oncol. Hematol..

[27] Ghattas N., Filice M., Abidi F., Guisàn J.M., Ben Salah A. (2014). Purification and improvement of the functional properties of *Rhizopus oryzae* lipase using immobilization techniques. J. Mol. Catal. B Enzym..

[28] Mateo C., Abian O., Bernedo M., Cuenca E., Fuentes M., Fernandez-Lorente G., Palomo J.M., Grazu V., Pessela B.C.C., Giacomini C. (2005). Some special features of glyoxyl supports to immobilize proteins. Enzym. Microb. Technol..

[29] Barbosa O., Ortiz C., Berenguer-Murcia A., Torres R., Rodrigues R.C., Fernandez-Lafuente R. (2014). Glutaraldehyde in bio-catalysts design: A useful crosslinker and a versatile tool in enzyme immobilization. RSC Adv..

[30] Bavaro T., Torres-Salas P., Antonioli N., Morelli C.F., Speranza G., Terreni M. (2013). Regioselective deacetylation of disaccharides via immobilized *Aspergillus niger* esterase(s)-catalyzed hydrolysis in aqueous and non-aqueous media. ChemCatChem.

[31] Chung R.T., Gale M., Polyak S.J., Lemon S.M., Liang T.J., Hoofnagle J.H. (2008). Mechanisms of action of interferon and ribavirin in chronic hepatitis C: Summary of a workshop. Hepatology.

[32] Mateo C., Grazù V., Palomo J.M., Lopez-Gallego F., Fernàndez-Lafuente R., Guisàn J.M. (2007). Immobilization of enzymes on heterofunctional epoxy supports. Nat. Protoc..

[33] Fernàndez-Lafuente R., Rosell C.M., Rodriguez V., Santana C., Soler G., Bastida A., Guisàn J.M. (1993). Preparation of activated supports containing low pK amino groups. A new tool for protein immobilization via the carboxyl coupling method. Enzym. Microb. Technol..

[34] HHpred-Homology Detection & Structure Prediction by HMM-HMM Comparison. https://toolkit.tuebingen.mpg.de/hhpred.

[35] Modeller. https://toolkit.tuebingen.mpg.de/modeller.

[36] Serra I., Serra C.D., Rocchietti S., Ubiali D., Terreni M. (2011). Stabilization of thymidine phosphorylase from *Escherichia coli* by immobilization and post immobilization techniques. Enzym. Microb. Technol..

[37] Temporini C., Bonomi P., Serra I., Tagliani A., Bavaro T., Ubiali D., Massolini G., Terreni M. (2010). Characterization and study of the orientation of immobilized enzymes by tryptic digestion and HPLC-MS: Design of an efficient catalyst for the synthesis of cephalosporins. Biomacromolecules.

[38] Bradford M.M. (1976). A rapid and sensitive method for the quantitation of microgram quantities of protein utilizing the principle of protein-dye binding. Anal. Biochem..

