# Supplementary material for: Immobilization of Neutral Protease from Bacillus subtilis for Regioselective Hydrolysis of Acetylated Nucleosides: Application to Capecitabine Synthesis
Source: Molecules. 2016 Nov 25;21(12):1621. doi: 10.3390/molecules21121621 (PMC6273923; doi:10.3390/molecules21121621)
Supplement: Supplementary file 1 [file molecules-21-01621-s001.pdf]

Supplementary Materials: Immobilization of Neutral Protease from *Bacillus subtilis* for Regioselective Hydrolysis of Acetylated Nucleosides: Application to Capecitabine Synthesis

Teodora Bavaro, Giulia Cattaneo, Immacolata Serra, Ilaria Benucci, Massimo Pregnolato and Marco Terreni

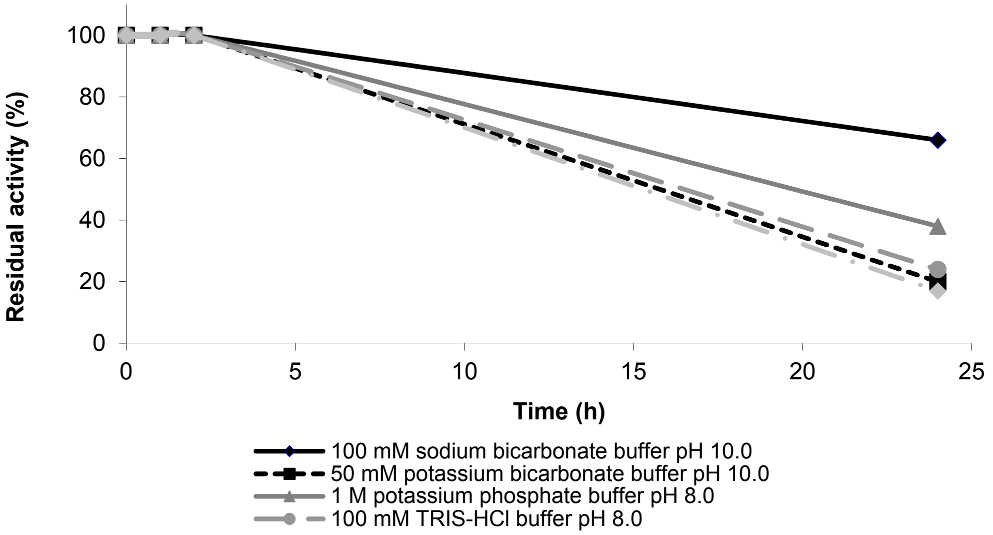

Figure S1. Stability of native protease N in experimental conditions of immobilization.

(**a**)

Sequence of *B. subtilis* neutral protease A

AAATGSGTTLKGATVPLNISYEGGKYVLRDLSKPTGTQIITYDLQNRQSRLPGTLVSSTTKTFTSSS
QRAAVDAHYNLGKVYDYFYSNFKRNSYDNKGSKIVSSVHYGTQYNN
AAWTGDQMIYGDGDGSFFSPLSGSLDVTAHEMTHGVTQETANLIYENQPGALNESFSDVFGYFN
DTEDWDIGEDITVSQPALRSLSNPTKYNQPDNYANYRNLPNTDEGDYGGVHTNSGIPNKAAYN
TITKLGVSKSQQIYYRALTTYLTPSSTFKDAKAALIQSARDLYGSTDAAKVEAAWNAVGL

Sequence of *S. aureus* metalloproteinase

AAATGTGKGVLGDTKDININSIDGGFSLEDLTHQGKLSAYNFNDQTGQATLITNEDENFVKDDQ
RAGVDANYYAKQTYDYYKNTFGRESYDNHGSPIVSLTHVNHYGGQDNRNNAAWIGDKMIYGD
GDGRTFTNLSGANDVVAHEITHGVTQQTANLEYKDQSGALNESFSDVFGYFVDDEDFLMGEDV
YTPGKEGDALRSMSNPEQFGQPSHMKDYVYTEKDNGGVHTNSGIPNKAAYNVIQAIGKSKSEQI
YYRALTEYLTSNSNFKDLKDALYQAAKDLYEQQTAEQVYEAWNEVGVE

(**b**)

```
Query  1    AAATGSGTTLKGATVPLNISYEGGKYVLRDLSKPTGTQIITYDLQNRQSRLPGTLVSSTT  60
            AAATG+G  + G T  +NI+   G + L DL+         ++ Q  Q+    TL+++
Sbjct  1    AAATGTGKGVLGDTKDININSIDGGFSLEDLTHQGKLSAYNFNDQTGQA----TLITNED  56

Query  61   KTFTSSSQRAAVDAHYNLGKVYDYFYSNFKRNSYDNKGSKIVSSVH---YGTQYNN---A  114
            + F    QRA VDA+Y   + YDY+ + F R SYDN GS IVS  H   YG Q N    A
Sbjct  57   ENFVKDDQRAGVDANYYAKQTYDYYKNTFGRESYDNHGSPIVSLTHVNHYGGQDNRNNAA  116

Query  115  WTGDQMIYGDGDGSFFSPLSGSLDVTAHEMTHGVTQETANLIYENQPGALNESFSDVFGY  174
            W GD+MIYGDGDG  F+ LSG+ DV AHE+THGVTQ+TANL Y++Q GALNESFSDVFGY
Sbjct  117  WIGDKMIYGDGDGRTFTNLSGANDVVAHEITHGVTQQTANLEYKDQSGALNESFSDVFGY  176

Query  175  FNDTEDWDIGEDITV---SQPALRSLSNPTKYNQPDNYANYRNLPNTDEGDYGGVHTNSG  231
            F D ED+ +GED+        ALRS+SNP ++ QP +  +Y       E D GGVHTNSG
Sbjct  177  FVDDEDFLMGEDVYTPGKEGDALRSMSNPEQFGQPSHMKDYVYT----EKDNGGVHTNSG  232

Query  232  IPNKAAYNTITKLGVSKSQQIYYRALTTYLTPSSTFKDAKAALIQSARDLYGSTDAAKVE  291
            IPNKAAYN I  +G SKS+QIYYRALT YLT +S FKD K AL Q+A+DLY    A +V
Sbjct  233  IPNKAAYNVIQAIGKSKSEQIYYRALTEYLTSNSNFKDLKDALYQAAKDLYEQQTAEQVY  292

Query  292  AAWNAVGL  299
             AWN VG+
Sbjct  293  EAWNEVGV  300
```

Figure S2. (**a**) Sequences of *B. subtilis* neutral protease A (Protease N) and *S. aureus* metalloproteinase, (**b**) Blast alignment between *B. subtilis* neutral protease A and *S. aureus* metalloproteinase sequences.

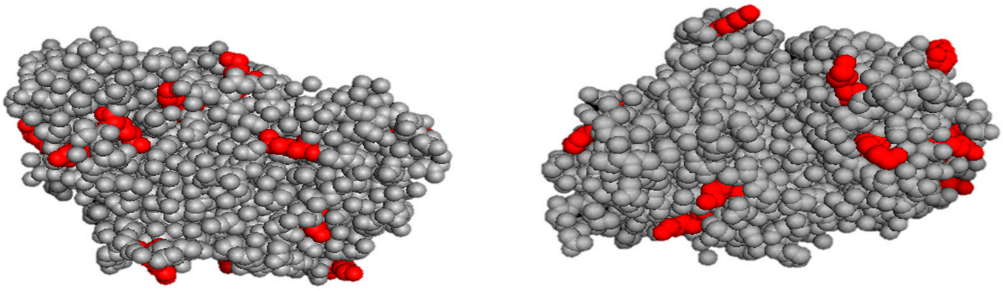

(**A**) (**B**)

Figure S3. Front (panel **A**) and back side (panel **B**) in the 3D structure of *S. aureus* metalloproteinase (pdb: 1qbq). Surface lysines are coloured in red.

Analytical Characterization of Compounds 1a and 5a

2,3-Di-O-Acetyluridine (**1a**): TLC (CH_2Cl_2/MeOH, 9:1): R_f = 0.51. ^{1}H-NMR (400 MHz, [D$_6$]DMSO, 25 °C): δ = 11.40 (s, 1 H, 3-NH), 7.89 (d, 1 H, 5-H), 6.00 (d, 1 H, 1-H), 5.70 (d, 1 H, 6-H), 5.50 (s, 1 H, OH in 5), 5.30 (m, 2 H, 2-H, 3-H), 4.14 (m, 1 H, 4-H) 3.64 (m, 2 H, 5-H) 2.10–2.02 (s, 6 H, 2 OAc) ppm. MS: calcd. for [M + 1]$^+$: 351.26; found 351.00.

2,3-Di-O-Acetylcytidine (**5a**): TLC (CH_2Cl_2/MeOH, 9:1): R_f = 0.50. ^{1}H-NMR (400 MHz, [D$_6$]DMSO, 25 °C): δ = 7.80 (d, 1 H, 5-H), 7.30 (s, 2 H, NH_2), 6.00 (d, 1 H, 1-H), 5.70 (d, 1 H, 6-H), 5.40 (m, 1 H, 2-H, 3-H, OH in 5), 4.10 (m, 1 H, 4-H), 3.70 (m, 2 H, 5-H), 2.10–2.00 (s, 6 H, 2 OAc) ppm. MS: calcd. for [M + Na]$^+$: 350.28; found 350.10.
